# Liver biopsy may facilitate pancreatic graft evaluation: Positive association between liver steatosis and pancreatic graft adipose infiltration

**DOI:** 10.6061/clinics/2018/e49

**Published:** 2018-05-19

**Authors:** Lucas S. Nacif, Vinicius Rocha-Santos, Laura C.L. Claro, Agustin Vintimilla, Leandro A. Ferreira, Rubens M. Arantes, Rafael S. Pinheiro, Wellington Andraus, Venancio A.F. Alves, Luiz Carneiro D’Albuquerque

**Affiliations:** I Divisao de Transplante de Figado e Orgaos do Aparelho Digestivo, Departamento de Gastroenterologia, Hospital das Clinicas HCFMUSP, Faculdade de Medicina, Universidade de Sao Paulo, Sao Paulo, SP, BR; II Departamento de Patologia, Hospital das Clinicas HCFMUSP, Faculdade de Medicina, Universidade de Sao Paulo, Sao Paulo, SP, BR

**Keywords:** Liver Transplant, Pancreas Transplant, Graft, Liver Steatosis, Visceral Fat

## Abstract

**OBJECTIVES:**

The number of pancreatic transplants has decreased in recent years. Pancreatic grafts have been underutilized compared to other solid grafts. One cause of discard is the macroscopic appearance of the pancreas, especially the presence of fatty infiltration. The current research is aimed at understanding any graft-related association between fatty tissue infiltration of the pancreas and liver steatosis.

**METHODS:**

From August 2013 to August 2014, a prospective cross-sectional clinical study using data from 54 multiple deceased donor organs was performed.

**RESULTS:**

Micro- and macroscopic liver steatosis were significantly correlated with the donor body mass index ([BMI]; *p*=0.029 and *p*=0.006, respectively). Positive gamma associations between pancreatic and liver macroscopic and microscopic findings (0.98; confidence interval [CI]: 0.95–1 and 0.52; CI 0.04–1, respectively) were observed. Furthermore, comparisons of liver microscopy findings showed significant differences between severe *versus* absent (*p*<0.001), severe *versus* mild (*p*<0.001), and severe *versus* moderate classifications (*p*<0.001). The area under the receiver operating curve was 0.94 for the diagnosis of steatosis by BMI evaluation using a cut-off BMI of 27.5 kg/m^2^, which yielded 100% sensitivity, 87% specificity, and 100% negative predictive value.

**CONCLUSIONS:**

We observed a positive association of macroscopic and microscopic histopathological findings in steatotic livers with adipose infiltration of pancreatic grafts.

## INTRODUCTION

The total number of pancreatic transplants performed in the United States has decreased since 2004 1,2. Pancreatic grafts have been underutilized compared to other solid organs, and the rates of pancreatic discard and non-procurement have increased 3. One cause of discard is a poor macroscopic appearance of the pancreas, mainly due to fatty infiltration of the organ.

Pancreatic graft adipose infiltration (PGAI) can be hazardous for whole pancreas transplant recipients. Since PGAI can increase postoperative complications, such as pancreatitis and pancreatic graft thrombosis, and cause a decrease in graft survival, many transplant teams discard the organ following macroscopic evaluation. However, macroscopic pancreatic examinations can be difficult due to the presence of peripancreatic adipose tissue, which leads to inaccurate evaluation of adipose infiltration into the pancreas 2,4,5. Pancreatic biopsy can be hazardous for pancreatic patients due to postoperative pancreatitis and fistula.

In contrast, liver graft steatosis is easily analyzed from both the macroscopic and microscopic points of view. Liver biopsy is very accurate 6, and steatosis can be classified as micro- or macrovesicular. Macrovesicular steatosis has a greater influence on ischemia/reperfusion injury and poor graft function than microvesicular steatosis, which occasionally occurs in isolated cases 5,6. During the organ recovery period, macrovesicular steatosis is generally suspected after the initial inspection. However, biopsy is the gold standard used to obtain an objective assessment 7. Furthermore, fatty tissue infiltration into pancreatic grafts does not undergo this type of evaluation.

Ninety percent of the organs used for whole organ pancreatic transplant are obtained from donors <50 years old with a body mass index (BMI) <30 kg/m^2^, while organs from older, more obese donors are more often recovered for islet transplantation or research 8. Another important factor observed when a pancreatic graft is refused is fatty tissue infiltration of the pancreas, which is the motivation for this study.

Our intention was to verify the association between PGAI and liver steatosis. We speculated that PGAI could be indirectly evaluated by liver graft analysis, thus avoiding organ discard and facilitating an evaluation of the pancreatic graft. The main objective of this study was to evaluate the association of the degree of fatty infiltration of the pancreatic graft with the degree of hepatic steatosis in multiple deceased donor organs.

## MATERIALS AND METHODS

### Study design

From August 2013 to August 2014, we analyzed clinical data from 54 multiple deceased donor organs in the Department of Gastroenterology at the University of Sao Paulo School of Medicine, Brazil. We prospectively studied, compared, and evaluated the associations among demographic, clinical, and laboratory data from deceased donors.

### Inclusion and exclusion criteria

All deceased liver donors that were transplanted in our service during the study period were included. To comply with the wishes of the ethical committee, we included graft cases not previously used for other reasons (age, BMI, comorbidities, drugs) to avoid interfering with the evolution of the graft. All members of the family of the deceased donor and the recipients agreed to the study protocol and provided informed consent.

### Donor technique definition

In all cases, we performed total hepatectomy en bloc in conjunction with the pancreas as the conventional organ recovery donor surgery. Preservation was performed using the University of Wisconsin solution (UW). We performed a liver and pancreatic biopsy, which was sent to the pathology department for microscopic study. A board-certified expert surgeon with extensive experience in pancreatic and liver transplant performed a macroscopic evaluation and classified the degree of liver steatosis and pancreatic liposubstitution as absent, mild (0%–30%), moderate (31%–70%), or severe (>70%). The associations between these factors were determined.

### Histopathology

**Figure 1:**
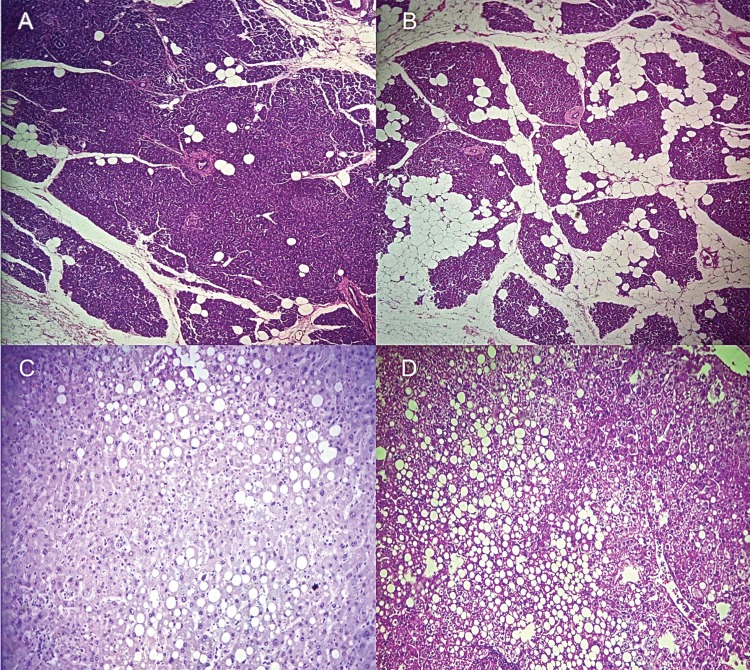
Illustration of microscopic pathologic findings: (A) mild and (B) moderate pancreatic graft adipose infiltration and (C) moderate and (D) severe steatotic liver.

Fifty-four segments of pancreatic tissue between 3.0 and 5.0 cm in diameter (with no transplanted pancreatic graft) and liver biopsy (with transplanted hepatic grafts) specimens were obtained using a Tru-Cut biopsy needle 14G (Langeskov, Denmark). The segments were fixed in 10% formalin and sent to the pathology division for macroscopic evaluation. Three fragments were chosen, thereby generating three blocks each per case. The material was processed and embedded in a paraffin mold until it had completely solidified. Four-micrometer sections were cut, placed on a slide, and stained with hematoxylin and eosin. The evaluation was performed by the same pathologist (a board-certified expert pathologist with extensive experience in hepatobiliary and liver transplant) without knowledge of clinical information and prior macroscopic evaluation of formaldehyde-fixed sections. Fatty replacements of the pancreatic parenchyma and liver steatosis were evaluated as pathological parameters (Figure 1).

### Statistical analysis

Median (25%–75% quantile) or mean and standard deviation values are presented for quantitative variables, and percentages are presented for categorical variables. Gamma ordinal association (strong >0.7, moderate <0.7 and >0.4, and low <0.4) was performed between categorical variables 9. Analysis of variance followed by the Tukey or Kruskal-Wallis test followed by the non-parametric Tukey test were applied to analyze differences among groups 10. Finally, a receiver operating characteristic (ROC) curve was used to determine cutoff points for examination values. All analyses were conducted with the R statistical program, version 2.15.1 11. A value of *p*<0.05 was considered statistically significant in the final analysis.

### Ethical aspects

The study was approved by the Institutional Review Ethics and Research (Cappesq) committee of the Hospital das Clinicas HCFMUSP, Faculdade de Medicina, Universidade de São Paulo, number 399.857, on 18/07/2013, fulfilling all requirements for studies on humans according to the guidelines of the 1975 Helsinki Declaration.

## RESULTS

### Clinical and demographic profile of the population

Clinical data from 54 deceased donors from August 2013 to August 2014 were studied. The mean donor age was 40.82±17.24 years, and the median age was 43 (ranging from 5 to 71) years. The mean donor BMI was 24.38±4.38, and the median was 24 (ranging from 15.3 to 31). Moreover, the surgeon evaluated macroscopic steatosis and adipose infiltration into the pancreas for the classification of subtypes as shown in Table 1.

The macroscopic evaluation of liver steatosis cases showed that steatosis was absent in 42.86%, mild in 39.29%, moderate in 14.29%, and severe in 3.57% of cases. Pancreatic macroscopic liposubstitution was absent in 36.36%, mild in 30.3%, moderate in 27.27%, and severe in 6.06% of cases.

The microscopic evaluation indicated no steatosis in 40%, mild steatosis in 42%, moderate steatosis in 10%, and severe in steatosis 8% of cases. Pancreatic microscopic liposubstitution was absent in 13.33%, mild in 80%, moderate in 10%, and severe in 8% of cases.

The demographic associations between the liver and pancreatic macroscopic and microscopic evaluations are shown in Figure 1, and multiple comparisons and analyses are shown in Table 2.

### Group comparisons and associations

Gamma ordinal association was performed between categorical variables. Table 3 shows a significant association between pancreatic and liver macroscopic findings (0.98; CI 0.95–1) and between pancreatic and liver microscopic findings (0.52; CI 0.04–1). Tests were applied to determine differences among groups, as shown in Table 3. The results in Table 2 demonstrate statistically significant associations of microscopic and macroscopic liver steatosis with donor BMI (0.029 and 0.006, respectively). Multiple comparison tests for variable donor BMI are shown in Table 4, which demonstrates a significant association of macroscopic liver steatosis between the moderate and absent groups (*p*=0.030). Furthermore, a comparison of liver microscopic findings showed significant differences between the severe *versus* absent (*p*<0.001), severe *versus* mild (*p*<0.001), and severe *versus* moderate groups (*p*<0.001), as shown in Table 4.

### ROC curve analysis

**Figure 2:**
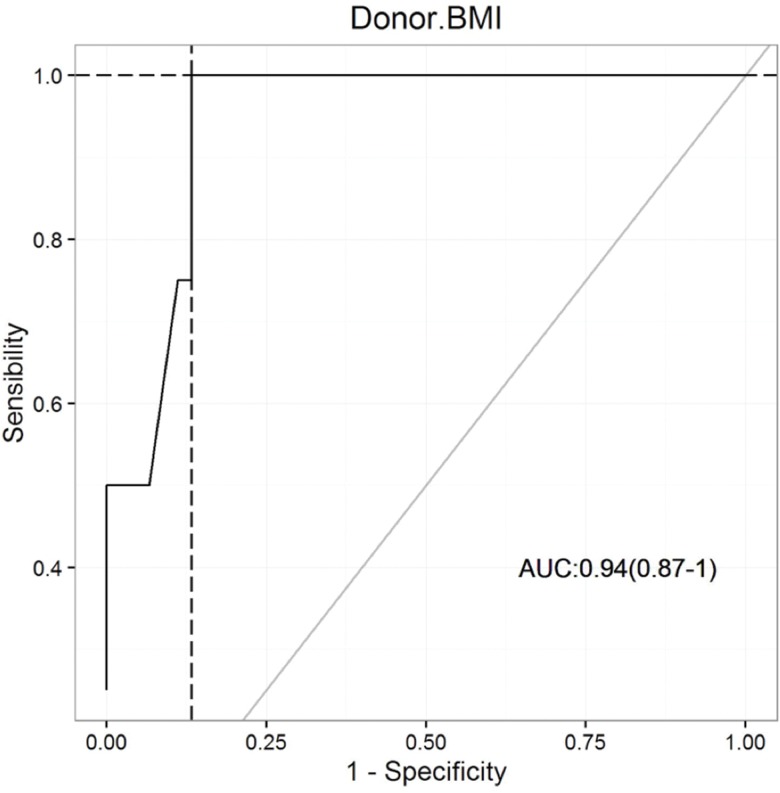
AUROC curve evaluation of the diagnosis of steatosis using BMI.

The area under the ROC curve (AUROC) of 0.94 demonstrates an excellent fit, as shown in Figure 2. This finding indicates an improved steatosis diagnosis corresponding to BMI evaluation. The ROC curve revealed an optimal BMI cut-off value of 27.5 kg/m^2^, with a sensitivity of 100%, specificity of 87%, negative predictive value 100%, and positive predictive value of 40% (Figure 2).

## DISCUSSION

In this study, we observed a positive association between macroscopic evaluation by a surgeon and microscopic pathologic findings in relation to steatotic liver and adipose infiltration of pancreatic grafts. Furthermore, we determined the optimal donor BMI value for predicting steatosis and adipose infiltration of the pancreatic graft.

Stratta et al. discussed several methods to alleviate the decrease in pancreatic transplants 2, including expanding the number of acceptable donors 2. The present study may facilitate the process of pancreatic transplant by improving the evaluation of the association between pancreatic graft and liver biopsy. We found a positive association between macroscopic and microscopic findings in relation to steatotic liver and adipose infiltration of the pancreatic graft. This finding indicates that macroscopic findings should be compared with the microscopic findings. Furthermore, pancreatic grafts do not have to be discarded based on possible mistakes by the surgeon who interprets the macro/microscopic results. In this situation, we suggest confirming the association of the pancreatic histopathology analysis with the liver biopsy.

A growing obesity epidemic coupled with a high diabetes prevalence in the general population can cause hepatic steatosis and likely causes fatty tissue infiltration of the pancreas 4. This is one of the donor characteristics that is termed “marginal.” This situation is increasingly common and interferes with graft procurement 4. Additional donors, based on recently expanded criteria, are desired due to organ shortages and a constant imbalance between available organs and transplant candidates. The literature shows that variations occur in the definitions, selection criteria, and use of expanded criteria donors according to different geographical areas and centers based on the acceptable risk of graft failure 4,5.

Cucchetti A et al. analyzed data from 374 deceased liver donors from whom a liver biopsy had been obtained to identify variables that could predict the degree of macrovesicular steatosis. Steatosis could be identified accurately to a level of >30% with an AUC of 0.86 (95% CI=0.81–0.91) in combination with BMI, an elevation of alanine aminotransferase, the presence of type II diabetes, a history of heavy alcohol consumption, and ultrasonographic steatosis signs 12. In the present study, we found that an AUROC curve of 0.94 provided an excellent tool for BMI-associated steatotic diagnosis. The AUROC curve area revealed an optimal BMI cut-off value of 27.5 kg/m^2^ with a sensitivity of 100%, specificity of 87%, and negative predictive value of 100%.

During organ recovery surgery, inspection at procurement facilitates detection of the degree of liver steatosis. However, a poor correlation exists between surgical assessment and the degree of steatosis when the degree of steatosis is >35%. A biopsy should then be systematically performed 5. In this study, difficulties were encountered in evaluating liver steatosis at advanced stages and performing correlations with severe fatty infiltration of the pancreas.

Criteria of liver texture (yellowness, round edges, and an absence of capsular scratch marks) were associated with and were more helpful in identifying macrosteatotic organs than the actual steatosis estimation by the surgeon 3. This important point could help surgeons to classify liver steatosis; however, in this study, we described a new classification of PGAI with subclassifications of absent, mild, moderate, and severe.

Improved allocation management also helps to adjust waiting time and establish a uniform pancreas allocation policy. Under current allocation policy, waiting time accrued by pancreas-alone candidates will not be considered in the listing for concurrent kidney-pancreas or kidney-alone transplants 8. Furthermore, rates of pancreatic graft acceptance have demonstrated a significant decrease in recent years (from 46.4% to 25% [*p*<0.05]), and despite greater organ availability, pancreatic transplants have remained stable 13. This study described new pancreatic macroscopic and microscopic evaluations of graft adipose infiltration aimed at improving organ acceptance and allocation.

This study had several limitations: 1) it was a single-center study with a relatively small sample size; 2) because this was a clinical practice study, all liver grafts that were transplanted were included; therefore, a few cases with severe steatosis or severe pancreatic infiltration, and 3) cross-sectional macroscopic and microscopic analysis of the pancreas and liver were performed. Furthermore, an important benefit of this study was obtaining information about the association between the degree of steatosis and fatty infiltration of the pancreatic graft in multiple deceased donor organs, which could result in better allocation of the pancreatic graft based on the liver biopsy. However, we did not evaluate the transplant outcome correlations.

In conclusion, positive associations of macroscopic and microscopic histopathological findings in steatotic livers and adipose infiltration of the pancreatic graft were observed.

## AUTHOR CONTRIBUTIONS

All authors have approved the final version of the manuscript. Nacif LS was responsible for the study conception and design, data collection and analysis, manuscript writing and interpretation, and literature search. Rocha-Santos V, Pinheiro RS, Vintimilla A, and Ferreira LA were responsible for the study conception and design, data collection and analysis, interpretation and critical revision of the manuscript. Alves VA, Claro LC and Arantes RM were responsible for the study conception, interpretation and critical revision of the manuscript. Andraus W and D’Albuquerque LC were responsible for the study conception, interpretation and critical revision of the manuscript.

## Figures and Tables

**Table 1:** Demographic parameters for all deceased donors evaluated.

Parameters	Deceased donor (n=54)
Sex (n/%)	M=34 (62.9%) / F=20 (37.1)
Mean age (years)	40.82±17.24
Median age (years)	43 (range, 5-71)
Mean donor BMI	24.38±4.38
Median donor BMI	24 (range, 15.3-31)
Macroscopic evaluation of liver steatosis (%/ n)	Absent 42.86% (N=23) Mild 39.29% (N=21) Moderate 14.29% (N=8) Severe 3.57% (N=2)
Pancreatic macroscopic liposubstitution (%/ n)	Absent 36.36% (N=20) Mild 30.3% (N=17) Moderate 27.27% (N=14) Severe 6.06% (N=3)
Microscopic evaluation of liver steatosis (%/ n)	Absent 40% (N=22) Mild 42% (N=23) Moderate 10% (N=5) Severe 8% (N=4)
Pancreatic microscopic liposubstitution (%/ n)	Absent 13.33% (N=5) Mild 80% (N=43) Moderate 10% (N=4) Severe 8% (N=2)

**Note:** BMI, Body mass index; Surgeon, macroscopy; Pathology, microscopy.

**Table 2:** Multiple comparison tests for all parameters and groups.

Parameters	Organ	Severity	Median (25%-75%)		*p*-value
			Surgeon	Pathology	
Donor age	Pancreas	Absent	40 (28 - 49.25)	33.5 (15.75 - 40)	0.399
		Mild	50.5 (34.75 - 57.75)	45 (34 - 54)	0.565
		Moderate	44 (31.5 - 58)	48 (35.5 - 57.5)	0.999
		Severe	54.5 (51.25 - 57.75)	-	-
	Liver	Absent	43.5 (28 - 50.5)	40 (18.25 - 52)	0.999
		Mild	49 (39 - 59.5)	42.5 (36.25 - 56)	0.517
		Moderate	53 (40.5 - 63.25)	48 (48 - 50)	0.902
		Severe	-	38 (30.25 - 45)	-
Donor BMI	Pancreas	Absent	21.8 (19.45 - 26.02)	21.6 (20.25 - 22.57)	0.707
		Mild	25.15 (24.18 - 26.85)	24.2 (22.7 - 27.2)	0.353
		Moderate	23.7 (21.08 - 24.65)	24.7 (23.75 - 24.9)	0.609
		Severe	25.6 (24.2 - 27)	-	-
	Liver	Absent	21.8 (19.45 - 26.1)	22.45 (19.73 - 24.88)	0.885
		Mild	24.7 (23.95 - 26.55)	24.2 (23.12 - 26.73)	0.411
		Moderate	27.2 (25.3 - 30.75)	25.1 (25.1 - 25.7)	0.268
		Severe	-	32.8 (27.73 - 37.85)	-

**Note:** Median and interquartile range (25%-75%); BMI, Body mass index; Surgeon, macroscopy; Pathology, microscopy.

**Table 3:** Gamma ordinal association between categorical variables.

Factor 01	Factor 02	Gamma	95% CI
Pancreas, macro	Pancreas, micro	0.04	[-0.44; 0.52]
Liver, macro	Liver, micro	0.18	[-0.33; 0.7]
Pancreas, macro	Liver, macro	0.98	[0.95; 1]
Pancreas, micro	Liver, micro	0.52	[0.04; 1]

**Note:** CI, confidence interval; Surgeon, macroscopy (macro); Pathology, microscopy (micro).

**Table 4:** Multiple comparison tests for donor body mass index variables.

Factor	Group comparison	%/n	*p*-value
Liver, surgeon	Mild *vs*. Absent	39.2 (n=21) *vs*. 42.8 (n=23)	0.189
	Moderate *vs*. Absent	14.2 (n=8) *vs*. 42.8 (n=23)	0.030
	Moderate *vs*. Mild	14.2 (n=8) *vs*. 39.2 (n=21)	0.349
	Mild *vs*. Absent	39.2 (n=21) *vs*. 42.8 (n=23)	0.313
	Moderate *vs*. Absent	14.2 (n=8) *vs*. 42.8 (n=23)	0.238
Liver, pathology	Severe *vs*. Absent	8 (n=4) *vs*. 40 (n=22)	<0.001
	Moderate *vs*. Mild	10 (n=5) *vs*. 42 (n=23)	0.999
	Severe *vs*. Mild	8 (n=4) *vs*. 42 (n=23)	<0.001
	Severe *vs*. Moderate	8 (n=4) *vs*. 10 (n=5)	<0.001

**Note:** BMI, Body mass index; Surgeon, macroscopy; Pathology, microscopy.
